# Draft Genome Sequence of Ochrobactrum anthropi Strain ML7 Isolated from Soil Samples in Vinhphuc Province, Vietnam

**DOI:** 10.1128/genomeA.00218-15

**Published:** 2015-04-02

**Authors:** Nicholas J. Tobias, Bagdevi Mishra, Deepak K. Gupta, Long Phan Ke, Marco Thines, Helge B. Bode

**Affiliations:** a Fachbereich Biowissenschaften, Merck Stiftungsprofessur für Molekulare Biotechnologie, Goethe-Universität Frankfurt, Frankfurt am Main, Germany; b Biodiversity and Climate Research Centre, Frankfurt, Germany; c Vietnam National Museum of Nature, Vietnam Academy of Science and Technology, Caugiay, Hanoi, Vietnam; d Buchmann Institute for Molecular Life Sciences, Goethe-Universität Frankfurt, Frankfurt am Main, Germany

## Abstract

Ochrobactrum species are widespread in the environment and can colonize a wide variety of habitats. Here, we describe the sequencing of a new environmental isolate of Ochrobactrum anthropi isolated from northern Vietnam.

## GENOME ANNOUNCEMENT

Ochrobactrum anthropi has been isolated from a variety of environmental samples and can cause potentially serious opportunistic and nosocomial infections (1, 2). Ochrobactrum is a member of the *Alphaproteobacteria*, and it is phylogenetically related to other soil pathogens and the human pathogens Brucella and Acinetobacter. Ochrobactrum has been described as a suitable symbiont for *Steinernema* development (3), a known host for other bacterial symbionts, such as Xenorhabdus species (4). This strain was isolated from the nematode species *Steinernema longicaudum* from soil in Melinh, Vinhphuc Province, Vietnam, using a Galleria mellonella baiting trap.

The strain was grown at 30°C in LB broth for at least 16 h. DNA was extracted using the DNeasy blood and tissue kit (Qiagen). This strain was sequenced at Eurofins Genomics (Ebersberg, Germany) using an Illumina HiSeq 2500 instrument with 150-bp paired-end reads. A total of 4,686,931 clusters were generated, with a total yield of 1,406 Mbp (281× coverage). A two-step quality control approach was used to ensure good-quality reads for assembly. First, Trimmomatic (version 0.32) was used to trim the attached adapters and low-quality bases from both ends of the reads. Further, reads having an average base quality of <30 or Ns in the sequence were discarded. Read pairs for which both forward and reverse reads passed the above-mentioned filters and were >90 bases were retained for assembly (873 Mbp, 174× coverage). *De novo* assembly was carried out using Velvet (version 1.2.10), using *k*-mer lengths between 71 and 89, and an optimal assembly was obtained, with a *k*-mer length of 77. After removing contigs <300 bases, the final assembly resulted in 75 scaffolds (117 contigs), with an *N*_90_ of 31. The 4,909,639-bp genome has a G+C content of 56.0% and contains 4,831 protein-coding sequences, 49 tRNAs, and one copy of each rRNA subunit. Mapping of contigs to the fully assembled O. anthropi ATCC 49188 genome using Projector2 (5) suggests that this strain also contains two chromosomes, but an analysis with PlasmidFinder 1.2 (6) failed to identify any known origins of replication despite some contigs mapping to two of the four plasmids identified in the previously sequenced strain (7).

Based on antiSMASH 2.0 (8) and ClusterFinder (9) analyses, 22 predicted secondary metabolite gene clusters were identified, including a predicted herboxidiene gene cluster, a class of compounds with described anticholesterol (10) and antitumor properties (11, 12). In addition, one siderophore, one microcin, one ectoine, one terpene, two predicted galactoglucan, four unknown fatty acid, 12 unknown saccharide, and one hybrid fatty acid-saccharide gene cluster were predicted. One β-lactamase gene was also found within the genome consistent with the previously sequenced O. anthropi genome (99.83% identity) (7). The diversity of predicted natural products might be important for the potentially diverse lifestyle of these bacteria.

### Nucleotide sequence accession numbers.

The whole-genome shotgun project of O. anthropi ML7 has been deposited in GenBank under the accession no. JYFX00000000. The version described in this paper is version JYFX01000000.
